# Personally Tailored Survival Prediction of Patients With Follicular Lymphoma Using Machine Learning Transcriptome-Based Models

**DOI:** 10.3389/fonc.2021.705010

**Published:** 2022-01-10

**Authors:** Adrián Mosquera Orgueira, Miguel Cid López, Andrés Peleteiro Raíndo, Aitor Abuín Blanco, Jose Ángel Díaz Arias, Marta Sonia González Pérez, Beatriz Antelo Rodríguez, Laura Bao Pérez, Roi Ferreiro Ferro, Carlos Aliste Santos, Manuel Mateo Pérez Encinas, Máximo Francisco Fraga Rodríguez, Claudio Cerchione, Pablo Mozas, José Luis Bello López

**Affiliations:** ^1^ University Hospital of Santiago de Compostela, SERGAS, Santiago de Compostela, Spain; ^2^ Health Research Institute of Santiago de Compostela, Santiago de Compostela, Spain; ^3^ University of Santiago de Compostela, Santiago de Compostela, Spain; ^4^ Istituto Tumori della Romagna Istituto Romagnolo per lo Studio dei Tumori (IRST) “Dino Amadori” - Istituto di Ricovero e Cura a Carattere Scientifico (IRCCS), Meldola, Italy; ^5^ Hospital Clinic de Barcelona, Barcelona, Spain

**Keywords:** machine learning, lymphoma, follicular, gene expression, survival

## Abstract

Follicular Lymphoma (FL) has a 10-year mortality rate of 20%, and this is mostly related to lymphoma progression and transformation to higher grades. In the era of personalized medicine it has become increasingly important to provide patients with an optimal prediction about their expected outcomes. The objective of this work was to apply machine learning (ML) tools on gene expression data in order to create individualized predictions about survival in patients with FL. Using data from two different studies, we were able to create a model which achieved good prediction accuracies in both cohorts (c-indexes of 0.793 and 0.662 in the training and test sets). Integration of this model with m7-FLIPI and age rendered high prediction accuracies in the test set (cox c-index 0.79), and a simplified approach identified 4 groups with remarkably different outcomes in terms of survival. Importantly, one of the groups comprised 27.35% of patients and had a median survival of 4.64 years. In summary, we have created a gene expression-based individualized predictor of overall survival in FL that can improve the predictions of the m7-FLIPI score.

## Introduction

Follicular Lymphoma (FL) is one of the most common types of lymphoma, accounting for 10% of all lymphoid neoplasms among European populations (1). FL is a low-grade malignancy which is mostly considered incurable. Watch-and-wait is the recommended strategy in patients with low-tumor burden, but most of these eventually progress and need treatment with rituximab-based immunochemotherapy schemes, which commonly achieve prolonged remission periods and high overall survival. Despite the indolent nature of the disease in many cases, its estimated 10-year mortality rate is 20%, which is mostly conditioned by lymphoma relapse, chemorefractoriness and transformation to higher grade histologies (2).

As new drugs are developed for FL (3), it becomes increasingly important to provide patients with an optimal prediction about the expected outcome of treatment with first line immunochemotherapy, as this would improve the quality of decision-making in the management of the disease. Today, risk stratification of FL is still based on the *Follicular Lymphoma International Prognostic Index* (FLIPI) (4). FLIPI is a five-factor risk model based on age, stage, lactate dehydrogenase, hemoglobin levels and number of involved nodal areas. This score has been validated as a prognostic model in both the pre- and post-rituximab eras. A modified version of this score (FLIPI2) has been proposed, which has rendered slightly improved results compared with the original FLIPI score (5). More recently, the PRIMA prognostic index (PRIMA-PI) has developed a new prognostic score in the context of the prospective clinical trial PRIMA, which tested Rituximab-chemotherapy treatments with or without Rituximab maintenance (6). This score was based solely on β2-microglobulin and bone marrow involvement, and was predictive of progression free survival. Notably, PRIMA-PI was correlated with progression of disease in the 24 months from first line treatment (POD24), which is a strong predictor of short overall survival (7). Recently, a modified version of FLIPI that includes lymphopenia as a covariate improved the original score and enhanced the capacity to detect POD24 patients (8).

Several attempts have been made to apply molecular data for FL outcome prediction. The improved molecular characterization of FL has led to the identification of new disease subtypes with prognostic implications. In this line, established a clinico-genomic model named m7-FIPI that included the mutation of 7 genes (*EZH2*, *ARID1A*, *MEF2B*, *EP300*, *FOXO1*, *CREBBP* and *CARD11*), the FLIPI score and the Eastern Cooperative Oncology Group (ECOG) performance status (9). This score was predictive of 5-year failure-free survival and outperformed the prognostic value of FLIPI and FLIPI plus ECOG models. Although m7-FLIPI was trained to predict event-free survival, the subgroups derived from this model were also predictive of overall survival. In another report, Huet et al. described a gene expression profiling (GEP) approach based on 23 genes which identified two groups of FL patients with remarkably different progression-free survival independently of rituximab maintenance and FLIPI score (10). However, this model was not predictive of overall survival.

In this line, the implementation of machine learning (ML) based survival models has become popular in order to provide patient-centered risk information. Recently, we demonstrated the feasibility of using information gathered from gene expression profiling and clinical data to make individual ML predictions about diffuse large B-cell lymphoma patient outcome (11). The objective of this work was to apply the same strategy in the field of FL in order to devise new transcriptome-based prognostic models predictive of overall survival that can improve state-of-the-art risk-stratification tools.

## Materials and Methods

### Data Source

Data produced Leich et al. based on a FL patient cohort published by Dave et al. were used for training a model of overall survival (12, 13). These data were downloaded from the Gene Expression Omnibus (GEO ID GSE16131). The dataset comprised 184 patients diagnosed between 1974 and 2001 and treated with a variety of chemotherapy-based schemes and autologous stem-cell transplantation, or followed with observation. 4 samples were discarded due to lack of complete survival data. Median follow-up was 6.54 years and median survival was 10.15 years. Gene expression was determined using the Affymetrix Human Genome U133 A and B arrays.

Data from Pastore et al. were used for model validation (GEO ID GSE66166) (9). This dataset comprised gene expression measures from 138 patients with symptomatic lymphoma (advanced-stage or bulky disease) ineligible for curative radiotherapy who were treated with six to eight cycles of R-CVP (rituximab 375 mg/m² plus cyclophosphamide 1000 mg/m², and vincristine 1·4 mg/m² on day 1, and prednisone 100 mg/day, days 1–5) every 3 weeks at the British Columbia Cancer Agency (BCCA). Complete survival data was available for 106 cases. Patients were collected between February 24, 2004 and November 24, 2009. From 2006 onwards, patients with at least a partial response received rituximab maintenance (375 mg/m² given every 3 months for a total of eight doses). Median follow-up was 5.81 years and median survival was not reached. Gene expression measures were determined with the Illumina HumanHT-12 WG-DASL V4.0 expression beadchip.

### Preprocessing

Affymetrix Human Genome U133 A and B arrays gene expression estimates were fused and rank normalized. Similarly, Illumina HumanHT-12 WG-DASL V4.0 expression beadchip were ranked normalized. Afterwards, probes were annotated to gene symbols, and the expression of each gene was defined as the average value of all the probes matching the same gene. Common genes between both arrays were selected, comprising a total set of 14,882 genes. Finally, we used the parametric adjustment model implemented in ComBat in order to adjust for platform-related batch effects (14).

### Variable Selection

We created univariate cox regression models of overall survival considering the expression of each gene in the training set. P-values were adjusted for multiple testing using the Benjamini-Hochberg method. As no gene was statistically associated with overall survival genome-wide (q-value <0.1), we decided to consider the top 100 genes with lower p-values.

### Random Forest Models of Survival

Random forests were used to model overall survival. Such types of algorithms can quantify the relative importance of each variable, and thus enable the filtering of low-importance variables for model reduction and performance improvement (15). We initially created 100 models of survival in the training cohort by iteratively incorporating the expression of each one of the top 100 genes in p-value ascending order. Bootstrapping without replacement was performed with the default *by.node* protocol. Harrel’s concordance index (c-index) was used to assess model’s discriminative power on the bootstrapped training set and on the test set. C-indexes reflect to what extent a model predicts the order of events in a cohort. C-indexes below 0.5 indicate poor prediction accuracy, c-indexes near 0.5 indicate random guessing and c-indexes of 1 represent perfect prediction. Out-of-bag survival curves in the training set reflect the estimated error, also called out-of-bag estimate. This is a method of measuring the prediction error of random forests models utilizing bootstrap aggregating (bagging). Bagging uses subsampling to create training samples for the model to learn from. Out-of-bag error represents the mean prediction error on each training sample X, using only the trees that did not have X in their bootstrapped sample.

Continuous rank probability score (CRPS) was calculated as the integrated Brier score divided by time, and it represents the average squared distances between the observed status and the predicted survival probability at each time point. CRPS is always a number between 0 and 1, being 0 the best possible result. Variable reduction was performed by iteratively removing those variables with low importance. Variable importance was calculated with the *vimp* function, and samples with negative or low weight (importance < 1 × 10^-4^) were iteratively removed.

The model with the highest c-index in the training set was used for validation in the independent test set. Survival prediction on the test cohort was performed using the *predict.rfsrc* function with default parameters. Finally, we iteratively optimized the *ntree*, *mtry* and *nodesize* parameters in order to optimize the performance of the model. Briefly, we initially checked optimal *ntree* values by tuning the value between 500 and 1500 in chunks of 25. Afterwards, we tuned the *mtry* parameter from values of 2 to 40 in chunks of 2. Finally, we tuned the *nodesize* parameter from 1 to 40 in chunks of 1. In each step, the best model was considered as that which minimized the c-index value in the training set. Finally, the *nsplit* parameter (integer value for number of random splits to consider for each candidate splitting variable) was automatically adjusted according to deterministic splitting.

### Survival Prediction Performance, Multivariate Regression and Comparison With m7-FLIPI

We tested survival predictions created at different follow-up periods: 1, 2, 5 and 10 years, as well as maximum follow-up (15.7 years). Similarly, we represented the survival curves of patients in the training and test sets stratified by 5-year survival predictions (medians and terciles). Additionally, as the test set incorporated the m7-FLIPI variable, we also evaluated the performance of the predictions according to m7-FLIPI values and patient age. Cox c-indexes and confidence intervals were calculated. Finally, proportional hazards assumptions were tested with Schoenfeld’s method.

## Results

### Variable Selection

Baseline characteristics of the patients included in both cohorts are represented in **Table 1**. In order to prioritize genes for machine learning models, we tested the association of each individual gene with overall survival (total genes: 14,882). 966 and 112 genes had association p-values < 0.05 and < 0.005, respectively (univariate regression; **Supplementary Table 1**). Nevertheless, none of these achieved statistical significance after multiple testing adjustment (q-values > 0.1). Therefore, we arbitrarily selected the top 100 genes for fitting machine learning models of survival.

**Table 1:** Baseline characteristics of the two cohorts. Adopted from Pastore et al. (9) and Dave et al. (13).

	GSE16131 Training set	GSE66166 Test set
**Age > 60**	35.5%	55.0%
**Male**	41.9%	55.0%
**ECOG > 1**	9.4%	15.0%
**Raised LDH**	22.9%	21.0%
**FL Grade 3B**	0.0%	0.0%
**Median follow-up**	6.6 years	6.7 years

### Random Forest Models of Survival

**Figure 1:**
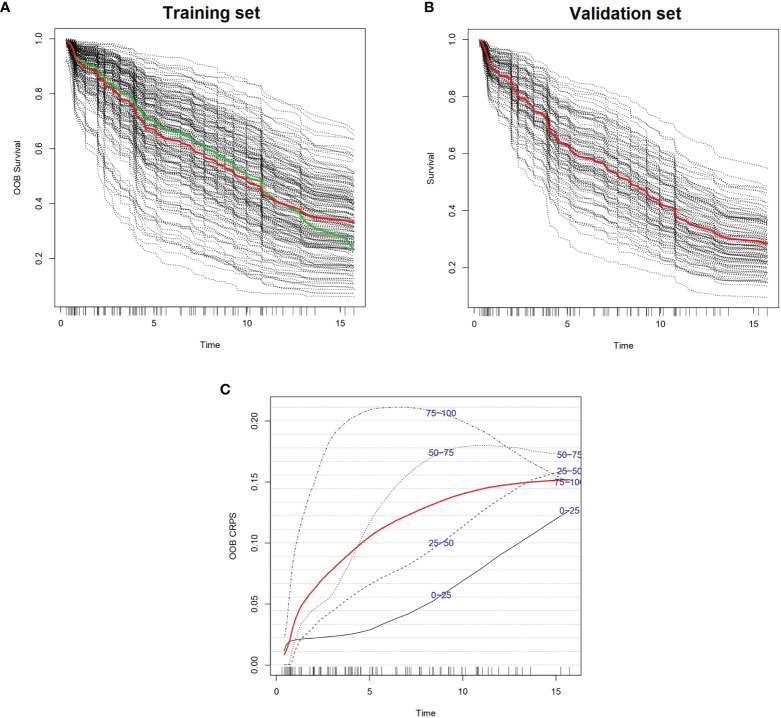
Predicted individual survival curves according to the most accurate random forest model (see text). **(A)** Out-of-bag survival curves predicted for patients within the training cohort (discontinuous black lines). The thick red line represents overall ensemble survival and the thick green line indicates the Nelson-Aalen estimator. **(B)** Individual survival curves predicted for patients within the test cohort (discontinuous black lines). The thick red line represents overall ensemble survival. Time scale is in years. **(C)** Representation of out-of-bag CRPS over time. Red line is the overall CRPS. Additionally, stratified CRPS by quarters of predicted ensemble mortality are provided. Vertical lines above the x axis represent death events.

We created 100 different random forest models of survival by iteratively adding one gene in p-value ascending order until reaching the top 100 genes. The gene with highest p-value was *RBM23* (p-value 4.62 x 10^-3^), and the gene with lowest p-value was *UBE2E1* (p-value 2.25 x 10^-5^). The best 10 models in terms of higher c-index were selected for variable reduction (**Supplementary Table 2**). Among these, the best model included 62 genes originating from the list of the top 96 genes (training set c-index 0.750, test set c-index 0.659). Parameter optimization of this model improved the model further, with c-indexes of 0.793 and 0.662 in the training and test cohorts, respectively (**Figure 1** and **Supplementary Table 3**). We named this model *Iacobus for Follicular Lymphoma* (IAC-FL). The most important variables in the model were the expression of *EMR1* and *C2orf73* (**Supplementary Table 4**).

### Model Predictions Over Time

**Figure 2:**
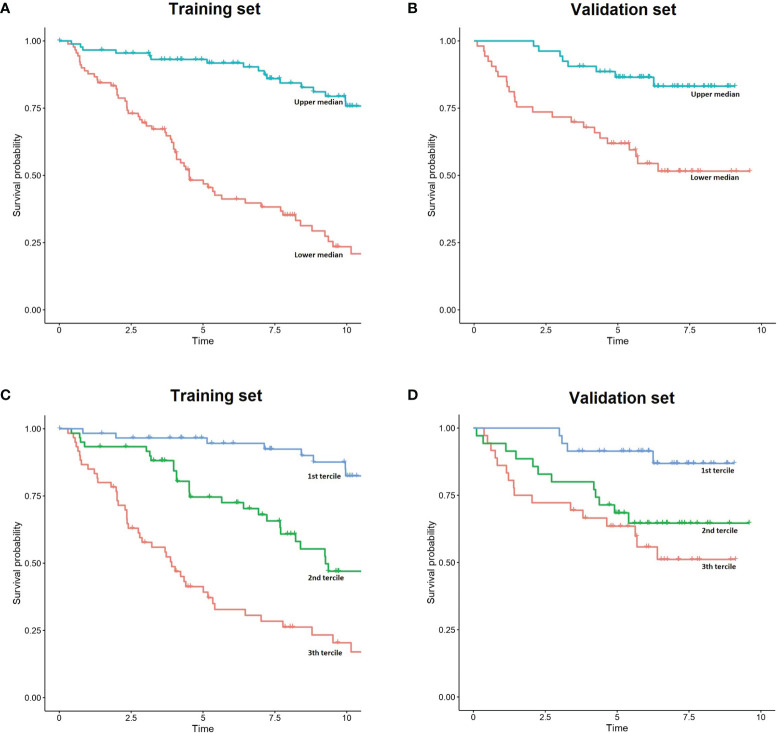
Simplified representation of IAC-FL predictions. **(A, B)** FL patient outcome according to the predicted 5-year survival medains created by IAC-FL in both the training and test sets. **(C, D)** Survival outcomes according to the predicted 5-year survival terciles created by IAC-FL in the training and test sets, respectively.

Survival predictions created by IAC-FL were significantly associated with real patient survival regardless of the follow-up time (**Table 2**). Cox models of survival considering predictions at several landmarks (1, 2, 5, 10 years and 15.7 years) indicate that such predictions were significantly associated with overall survival at all time points in both the training and the test cohorts, and these could identify groups with remarkably different outcomes (**Figure 2** and **Supplementary Table 5**). The lowest hazard ratios (HR) were achieved using 1-year predictions. In the same line, although the best c-indexes were achieved after 10 years of follow-up, good predictive accuracies in both cohorts were obtained even at 1 and 2 years of follow-up.

**Table 2:** C-indexes of the predictions created by IAC-FL at different time points in the training and test sets.

	Training cohort	Test Cohort
**1 year**	73,57	62,62
**2 years**	74,49	63,1
**3 years**	75,63	64,65
**4 years**	76,34	65,13
**5 years**	77,15	65,35
**6 years**	77,64	65,76
**7 years**	78,15	65,87
**8 years**	78,42	66,27
**9 years**	78,6	66,31
**10 years**	78,82	66,24
**11 years**	79,16	66,35
**12 years**	79,3	66,42
**13 years**	79,57	66,42
**14 years**	79,71	66,05
**15 years**	79,71	66,05
**Full follow-up**	79,35	66,16

In the test set, overall survival predictions created by the model at 1, 2, 5, 10 and 15.7 years were significantly associated with failure free survival (FFS) (**Supplementary Table 6**). Considering 5-year survival predictions, we stratified patients in the top and low 50% sets of expected survival (**Supplementary Figure 1**). Consistently, 37.73% of patients in the high-risk cluster experienced relapse or death due to FL within 2 years after first-line treatment, but this occurred only in 15.09% of those patients assigned to the low-risk cluster.

### Integration of IAC-FL Predictions With m7-FLIPI

Survival predictions created by IAC-FL at different time points were independent of m7-FLIPI score and absolute age (in years) in the test set (multivariate cox regression p-value < 0.05, **Supplementary Table 6**). The most significant findings were observed using longer follow-up predictions: p-values 3.11 x 10^-3^ and 3.01 x 10^-3^ & HR 5.49 x 10^-2^ & 9.48 x 10^-3^ at 10 and 15.7 years, respectively. Nevertheless, predictions at earlier time points were also significant: p-values of 0.03 and 0.01 & HR 2.79 x 10^-3^, 2.10 x 10^-3^ using 1 and 2-year survival predictions, respectively.

**Figure 3:**
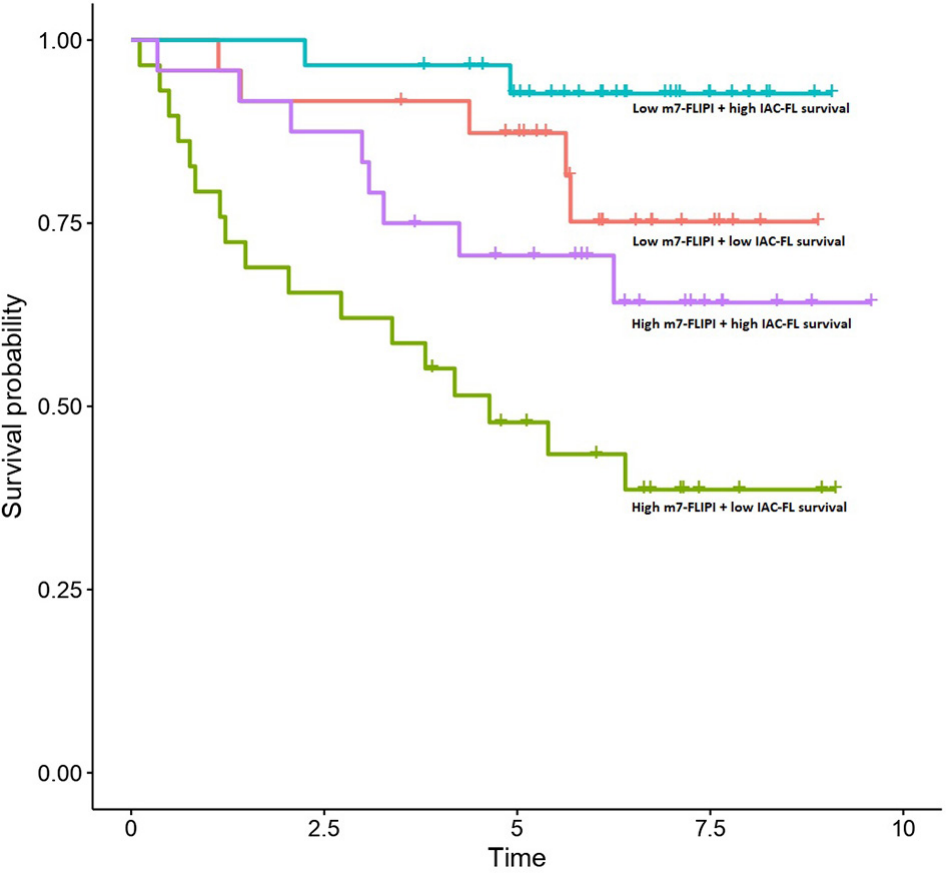
Survival outcomes of patients in the test set according to m7-FLIPI and IAC-FL predictions at maximum follow-up. Patients are stratified according to the medians of both variables.

Importantly, multivariate cox regression indicated that the integration of the predictions created by IAC-FL with m7-FLIPI and absolute age were superior to any of them separately, reaching a c-index of 0.794 (standard error, 0.035) when considering IAC-FL maximum-follow up predictions (15.7 years). These results are superior to those of the m7-FLIPI only (c-index 0.740 and standard error 0.053) and m7-FLIPI plus age models (c-index 0.749, standard errors 0.053). By stratifying IAC-FL’s predictions at 15.7 years and m7-FLIPI scores by their respective medians, 4 different groups with different survival could be observed (**Figure 3**). Importantly, we devised a group of 27.35% of patients with short median survival (4.64 years) after first line treatment. This subgroup was defined by m7-FLIPI scores above the median and IAC-FL survival predictions below the median.

## Discussion

In the present work, we present a ML predictor of overall survival in FL based on gene expression profiling named *IAC-FL*. The integration of this tool with m7-FLIPI enabled the identification of a patient subgroup at high risk of death in the first 5 years after first line immunochemotherapy. In contrast with other approaches (4–8), IAC-FL has been designed to directly predict overall survival rather than progression-free survival.

The identification of high-risk FL patients is key in order to guide novel therapeutic approaches and limit toxicity to low-risk cases. Some strategies have been developed to identify POD24 cases, as this subgroup is characterized by high mortality rates (5-year overall survival of 50%) (7). The m7-FLIPI model has 61% sensitivity and 79% specificity to identify POD24 patients, but a relevant proportion of patients who develop POD24 were classified as low risk. A different model, termed POD24-PI, has been developed by the same team in order to directly predict POD24, which achieved higher sensitivity at the expense of lower specificity and accuracy (16). Additionally, it is important to note that effective therapeutic approaches have been devised for POD24 patients, such as autologous hematopoietic stem cell transplantation or obinutuzumab plus lenalidomide combinations (17, 18). Therefore, risk-stratification models that directly intend to predict overall survival instead of progression free survival will enable a more precise identification of patients who are at high risk of lymphoma-related death from the moment of diagnosis or first-line treatment. In this line, we could easily identify a group of 27.35% of patients with median survival of 4.64 years in the era of rituximab. Therefore, integration of m7-FLIPI with IAC-FL is a promising strategy to provide overall survival personally tailored predictions in the real world.

Many of the genes included in the prognostic pattern are involved in cancer pathways. For example, *TMEM30A* loss-of-function mutations are correlated with B-cell receptor hyperactivation. These mutations drive B-cell lymphomagenesis and sensitize tumor cells to anti-CD47 “don’t eat me” blockage (19). *MAP4K4* is a member of the serine/threonine protein kinase family, and specifically activates MAPK8/JNK. The oncogenic functions of *MAP4K4* have been described in a variety of tumors, and selective inhibitors are being tested for cancer treatment (20). Similarly, *SETD2* is a histone methyltransferase that is specific for lysine-36 of histone H3, and methylation of this residue is associated with active chromatin. Coherently, *SETD2* gene is recurrently inactivated in a variety of B and T cell lymphomas (21, 22). Additionally, some genes such as *GZMH* and *CD247* are characteristically expressed in T-cells and others such as *EMR1* are expressed in macrophages. These findings are concordant with the functional and prognostic role of tumor microenvironment in FL (23). Overall, these findings indicate that the gene expression profile recapitulates the activity of important pathways in cancer, and particularly in lymphomagenesis, providing increased biological plausibility to the model.

Our approach has some limitations that must be considered. Firstly, there are some differences in patient characteristics between the training and test cohorts that might explain the differences in performance observed between both cohorts. Whereas those patients in the training set were treated in the pre-rituximab era or maintained in observation without treatment; patients in the test set were symptomatic (advanced-stage or bulky disease) and treated with homogeneous rituximab-based regimens. Additionally, the gene expression profiles were derived from different gene expression array platforms, and although we applied bioinformatic approaches to foster inter-study comparability, technical heterogeneity between cohorts probably exists. Taking these issues into consideration, it is reasonable to believe that the reproducibility of this predictor in homogenous cohorts analyzed with similar gene expression tools should be higher. Another issue that must be considered is that our comparison with m7-FLIPI could not discriminate between the prognostic value of the clinical variables and the mutations introduced in this score. This would be interesting to explore in future analysis, as machine learning models in other hematological cancers reveal that mutations add little prognostic benefit to gene expression profiles (24).

In comparison with other tools, this predictor has the advantage of making individualized predictions that do not reside in pre-established clinical and molecular subgroups, overcoming the limitations of imperfect patient subgrouping scores. Such an approach could drive the development of new first-line therapeutic interventions for selected high-risk patients based on personalized predictions. This strategy could involve the anticipated use of obinutuzumab-based combinations (25), which are more effective than standard rituximab-based schemes, or the administration of chemotherapy-free regimens based on rituximab plus lenalidomide, which have been tested as first-line therapy in small trials with encouraging results (26).

In summary, we have created and validated a transcriptomic predictor of overall survival in FL. The new classifier was independent of the m7-FLIPI score and absolute age, enabling an improved risk stratification of FL. It is necessary to standardize this prognostic tool in order to facilitate its incorporation in the clinical setting and to analyze its behaviour in association with other prognostic scores and different treatment schemes. Such approaches will bring *à la carte* treatment and survival predictions from bench to bedside.

## Data Availability Statement

The original contributions presented in the study are included in the article/**Supplementary Material**. Further inquiries can be directed to the corresponding author.

## Author Contributions

AMO had the idea, and performed the research. AMO, MCL, APR, AAB, JADA, MSGP, and BAR analyzed the results, wrote the paper. LBP, RFF, CAS, MMPE, MFFR, CC, PM, and JLBL made coments, gave final consent for publication. All authors contributed to the article and approved the submitted version.

## Funding

Article processinge charges have been payed with funds from the Fundación Galega de Hematoloxía e Hemoterapia.

## Conflict of Interest

AO has received honoraria for lectures and participation in advisory boards from Janssen and AstraZeneca. AO has received research grants from Roche and Celgene-BMS. JL has received honoraria for lectures and participation in advisory boards from Janssen, Abbey and Roche. JL has received research funds from Roche and Celgene-BMS.

The remaining authors declare that the research was conducted in the absence of any commercial or financial relationships that could be construed as a potential conflict of interest.

The reviewer GS declared a shared affiliation with one of the authors CC, to the handling editor at time of review.

## Publisher’s Note

All claims expressed in this article are solely those of the authors and do not necessarily represent those of their affiliated organizations, or those of the publisher, the editors and the reviewers. Any product that may be evaluated in this article, or claim that may be made by its manufacturer, is not guaranteed or endorsed by the publisher.

## Supplementary Material

Supplementary Table 1 Association between the expression of the top 100 genes and overall survival (univariate cox regression).

Supplementary Table 2 C-indexes of the 100 models trained with the top 100 genes in p-value descending order (training cohort).

Supplementary Table 3 Results of tuning the *ntree*, *mtry* and *nodesize* parameters in the best random forest model. C-indexes are referred to the training set.

Supplementary Table 4 Variables included in IAC-FL, ordered by decreasing importance.

Supplementary Table 5 Univariate cox regression of IAC-FL overall survival predictions at different time points in the training and test sets.

Supplementary Table 6 Univariate cox regression of IAC-FL failure-free survival predictions at different time points in the test set.

Supplementary Table 7 Multivariate cox regression of IAC-FL survival predictions at different time points in the test set. Covariates included in the model were absolute age and m7-FLIPI scores.

Supplementary Figure 1 Failure-free survival of patients in the test set stratified according to their overall survival predictions at 5 years. The 50% with higher expected survival is represented in blue, and the 50% with lower expected survival is represented in red.
