# Supplementary figures and images for: Personally Tailored Survival Prediction of Patients With Follicular Lymphoma Using Machine Learning Transcriptome-Based Models
Source: Front Oncol. 2022 Jan 10;11:705010. doi: 10.3389/fonc.2021.705010 (PMC8784530; doi:10.3389/fonc.2021.705010)

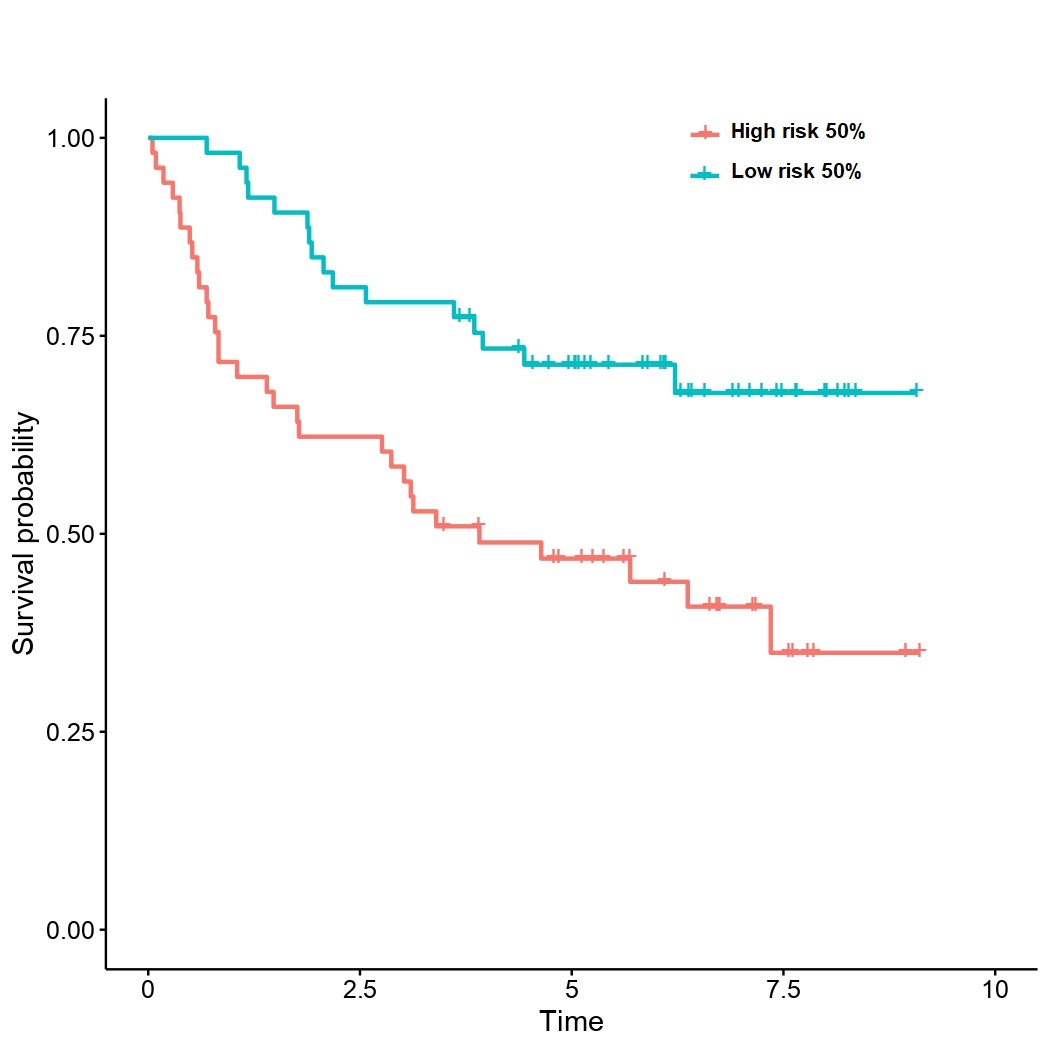

Supplement: Supplementary Figure 1 — Failure-free survival of patients in the test set stratified according to their overall survival predictions at 5 years. The 50% with higher expected survival is represented in blue, and the 50% with lower expected survival is represented in red. [file Image_1.jpeg]
